# Supplementary material for: Evaluating and redesigning the teaching practices of the Kazakh Mythology- engraving techniques
Source: PLoS One. 2024 May 14;19(5):e0296905. doi: 10.1371/journal.pone.0296905 (PMC11093332; doi:10.1371/journal.pone.0296905)
Supplement: Attachment — Submitted filename: Response to Reviewers.docx [file pone.0296905.s001.docx]

**Dear Reviewers,**

Subject: Response to Reviewer Comments and Gratitude

I hope this letter finds you well. I am writing to express my sincere gratitude for the invaluable feedback and constructive criticism you provided on my paper. I am immensely thankful for the time and effort you invested in reviewing my work, as it has significantly improved the quality and rigor of the manuscript.

I am pleased to inform you that I have carefully considered each of your suggestions and comments and made extensive revisions to address the concerns raised during the review process. Below, I summarize the major changes made in response to your insightful feedback:

Manuscript’s PLOS ONE's style requirement- Completed Reference style, Figures, Tables all formats corrected.

Research Data completely Available, can be requested at [karimbaigutov@gmail.com](mailto:karimbaigutov@gmail.com).

Dear Reviewer #1,

Thank you for your thorough and insightful review of our paper. We truly appreciate the time and effort you have invested in providing valuable feedback to enhance the quality of our manuscript. We have carefully considered each of your comments and have made the necessary revisions accordingly. Below, we address the changes we have made in response to your feedback:

1. Page 7: **The sentence is corrected** "Teaching Kazakh mythology arises several challenges among these challenges are the problems associated with students' engagement" to improve clarity.

Developing students’ interest in Kazakh mythology is a challenging task [5, 6]. One possible solution for developing students’ interest in Kazakh mythology is the use of engraving techniques. Through the engraving techniques students’ interests can be developed as introducing

2. Page 7: **The sentence is divided and corrected** "The first stage of the art of engraving is the exploration of the space of myth with the advent of human civilization, cultural landscapes begin to form, in which myth becomes..." into two separate sentences to improve readability and coherence.

The art engraving techniques evolve around the core concept of myth. The concept of myth can be considered among the oldest methods of explaining different social phenomena. Furthermore,

3. Page 7: **The sentence is corrected** and the reference inconsistency for the Slavic characters (Алтаев, & Иманбаева, 2021) and ensured that it matches the format used in the References section.

]. Methodological perspectives of Kazakh art, history, culture, and society as a whole assist in the interpretation of Kazakh myths and mythologies [12]

4. Page 8: **We have added a colon after the preposition "IN"** to improve grammatical accuracy. The revised sentence now reads: "regardless of which 'mental environment' they arise in: 'traditional and archaic', 'Hellenistic', or 'bourgeois'."

The period emphasizes the universality of the functions of myths, regardless of which "mental environment" they arise in,

5. Page 8: **We have corrected the grammar in the sentence:** "educators can bring by introduce students to the..." to ensure proper usage of the Gerund and phrasal verb.

Students’ engagement can be obtained by introducing Kazakh mythology as a main course in the curriculum. This may include a brief history of the art engraving techniques, along with an overview

6. Page 8: **We have ensured that the fonts used for the references** (Alexander, 2017; Slattery, 2005) match the entire text.

7. Page 8: **We have rephrased the sentence that lacked sense and improved its clarity**: "The pulsation and mutual permeability of 'logical rationalistic' and 'mythological' consciousness lead political elites to consider myths as an inexhaustible resource, a means of periodically renewing national identifications, and legitimizing certain forms of statehood and personal manifestations of power."

The concept of myth is usually based on “rational findings” and/or “fictional consciousness” Additionally, in the medieval period the concept of myths are often used as a source for the manifestation of power by the elites

8. Page 8: **We have provided more context and clarified**. "Naidysh" as a scholar to avoid any confusion.

20]. The neo-mythology is a new edition of mythology XIX century, neo-mythology surfaced as a result of the new understanding of the myth and its role in modern society. The myths fall into the domain of the “Quasi-scientific” and through the quasi-scientific approach, the fundamentals of myths can be explored.

9. Page 9: **We have revised the sentence to improve its structure and cohesion with the previous sentence**: "The purpose of this study was to identify the peculiarities of students' perception of Kazakh myths, assess their level of knowledge of mythology, identify the main problems in mastering the topic, and outline ways to study myths in teaching Kazakh mythology through the art of engraving."

This study was designed to explore students’ engagement and performance in an experiment designed to promote Kazakh mythology using engraving techniques at the Institute of Arts, Culture, and Sports, Abai University- Kazakhstan.

10. Page 9: **We have corrected the plural form of the noun to read** "60 Credits."

11. Page 9: We have replaced the verb with "**gain**," and the sentence now reads: "to gain students' interests."

12. Page 9: **We have rephrased the sentence to ensure proper subject-verb agreement and structure:** "The study was designed through the assessment of works by art historians and artists, analyzing programs and textbooks on art, mythology, graphics, and, of course, engraving to develop course content. The effectiveness of teaching Kazakh mythology through the art of engraving was evaluated using two groups of students from the art department."

The students’ centered research design was adopted to explore students’ interest in mythology and art engraving techniques. Furthermore, two study groups were formulated in the study,

13. Page 10: We have inserted a space to read "1 (strongly disagree)."

14. Page 10: We have inserted a space to read "1 - Student's engagement."

15. Page 11: We have removed the comma and ensured the proper use of third person singular: "Table 1 provides regression ..."

16. Page 11: We have used the passive voice: "that is explained by the independent variables."

17. Page 12: We have deleted the comma: "Table 2 shows..."

18. Page 13: We have addressed the tautology issue: "reducing the level of difficulty."

19. Page 13: We have corrected the sentence: "We recommend students use the works."

20. Page 13: We have deleted the comma: "The topics included in the curriculum consist of mythology and ..."

21. Page 13: We have reconsidered the noun usage: "the techniques of execution"

22. Page 13: We have corrected "Kazak" to read "Kazakh" consistently throughout the paper.

Additionally, we have thoroughly reviewed and improved the English writing, addressing extra-long sentences to ensure clarity and coherence.

Once again, we sincerely thank you for your meticulous review, which has undoubtedly contributed to the enhancement of our paper. Your valuable feedback has been instrumental in refining our work, and we are grateful for your commitment to maintaining the quality of research in our field.

If you have any further comments or suggestions, please do not hesitate to share them. We eagerly await your final assessment of our revised manuscript.

With utmost appreciation and regards,

Dr. Karim
